# Human immunodeficiency virus case detection and antiretroviral therapy enrollment among children below and above 18 months old: A comparative analysis from Cameroon

**DOI:** 10.1097/MD.0000000000025510

**Published:** 2021-04-30

**Authors:** Habakkuk A. Yumo, Jackson N. Ndenkeh, Isidore Sieleunou, Denis N. Nsame, Pius B. Kuwoh, Marcus Beissner, Thomas Loscher, Christopher Kuaban

**Affiliations:** a R4D International Foundation, Yaoundé; b Ludwig Maximilian University, Munich, Germany; c University of Montreal, Montreal, Canada; d Abong-Mbang District Hospital, Abong-Mbang; e Limbe Regional Hospital, Limbe; f University of Bamenda, Bamenda, Cameroon.

**Keywords:** adolescent, child, human immunodeficiency virus infection, human immunodeficiency virus testing, infants, polymerase chain reaction

## Abstract

While pediatric human immunodeficiency virus (HIV) testing has been more focused on children below 18 months through prevention of mother to child transmission of HIV (PMTCT), the yield of this approach remains unclear comparatively to testing children above 18 months through routine provider-initiated testing and counselling (PITC). This study aimed at assessing and comparing the HIV case detection and antiretroviral therapy (ART) enrolment among children below and above 18 months of age in Cameroon. This information is required to guide the investments in HIV testing among children and adolescents.

We conducted a cross-sectional study where we invited parents visiting or receiving HIV care in 3 hospitals to have their children tested for HIV. HIV testing was done using polymerase chain reaction (PCR) and antibody rapid tests for children <18 months and those ≥18 months, respectively. We compared HIV case detection and ART initiation between the 2 subgroups of children and this using Chi-square test at 5% significant level.

A total of 4079 children aged 6 weeks to 15 years were included in the analysis. Compared with children <18 months, children group ≥18 months was 4-fold higher among those who enrolled in the study (80.3% vs 19.7%, *P* < .001); 3.5-fold higher among those who tested for HIV (77.6% vs 22.4%, *P* < .001); 6-fold higher among those who tested HIV+ (85.7% vs 14.3%, *P* = .24), and 11-fold higher among those who enrolled on ART (91.7% vs 8.3%, *P* = .02).

Our results show that 4 out of 5 children who tested HIV+ and over 90% of ART enrolled cases were children ≥18 months. Thus, while rolling out PCR HIV testing technology for neonates and infants, committing adequate and proportionate resources in antibody rapid testing for older children is a sine quo none condition to achieve an acquired immunodeficiency syndrome (AIDS)-free generation.

## 1 Introduction

The human immunodeficiency virus/acquired immunodeficiency syndrome (HIV/AIDS) pandemic remains a global health threat. According to the UNAIDS estimates, 37.9 million people were living with HIV/AIDS globally in 2018, including 1.7 million children (<15 years). In that same year, 770,000 persons died of HIV/AIDS in the world, including 100,000 children. Africa hosts 68% of people living with HIV/AIDS (PLHIV) worldwide and in 2018, 62% of AIDS related deaths were from this continent.^[1]^ Despite this burden, transformative and remarkable progress has been made in the past decades with regards to the expansion of antiretroviral therapy (ART).^[2–4]^ In 2018, there were 23,300,000 PLHIV receiving ART, representing 62% of patients in need of treatment. At global level, this treatment expansion has contributed in the reduction of HIV incidence and mortality by 16% and 33%, respectively, from 2010 to 2018.^[1]^ However, children are not benefiting enough from this treatment expansion as there were 54% children on ART against 62% of adults as of June 2019.^[5]^ Cameroon (West and central Africa) with an HIV prevalence of 3.6% has 540,000 PLVIH, including 43,000 children. In 2018, 14,000 people died of AIDS in this country, including 3600 children. The gap in pediatric ART is even wider in Cameroon as 24% of eligible children are on treatment against 55% of adults.^[6]^

Multiple factors account for current gaps in pediatric and adolescent ART coverage. However, poorly-implemented and/or ineffective case-finding strategies are major barriers to ART roll out in this sub-population.^[7–9]^ More importantly, though achieving an AIDS-free generation requires the effective implementation of both prevention and treatment HIV services,^[10]^ the global agenda in the past decades has been focused more but on prevention. In this regard, the prevention of mother to children transmission of HIV (PMTCT) program has received more attention and resources from donors, policymakers, practitioners.^[11]^ Though the fourth prong of PMTCT include the provision of treatment to HIV-infected infants, this program provides services to infants only from birth up to 18 months.^[12]^ Consequently, HIV testing and treatment services for older children (≥18 months) has received less attention in the past decades. Actually, out of the PMTCT program, older children (≥18 months) are expected to be screened for HIV in health facilities through the WHO routine provider-initiated counseling and testing (PITC).^[13]^ Unfortunately, PITC implementation has been suboptimal across countries and this for several reasons including fear of stigma among parents/caregivers, lack of staff training, lack of HIV testing kits, poor commitment from facility leadership, and missed parental consent to test children.^[14–16]^ These weaknesses have left many HIV infected children undiagnosed and untreated in the community to the extent that pediatric HIV has been considered as neglected disease by some authors.^[17]^

The target population for pediatric HIV infection are children from 0 to 15 years.^[1]^ Thus, achieving an AIDS free generation requires investing adequate resources in HIV testing and treatment across this age group. As per WHO guidelines, children <18 months (in PMTCT) are HIV tested using polymerase chain reaction (PCR) while those older than 18 months (in PITC) are tested using antibody rapid tests.^[18]^ While more focus and emphasis have been on PCR testing, the yield of this approach in terms of pediatric HIV case detection and ART initiation compared with antibody rapid tests remain unclear. This study intends to bridge this scientific gap and provide information needed to guide pediatric HIV testing investments. This should contribute in reducing the current gap in HIV treatment for children in Cameroon and beyond.

## 2 Methods

### 2.1 Design

This was a cross-sectional study, part of the “Active Search for Pediatric HIV/AIDS” (ASPA) project published elsewhere.^[19]^ In the ASPA study, we invited all HIV infected parents receiving HIV care in 3 hospitals in Cameroon to have their children of unknown HIV status aged 6 weeks to 19 years tested for HIV (tPITC group). In the same hospitals, all parents/guardians who accompanied their sick children of the same age group for consultation at the outpatient departments were also counseled, and these children were invited to test for HIV irrespective of the presenting complaint (bPITC group).

### 2.2 Setting

The ASPA study was implemented at 3 public health facilities: Limbe Regional Hospital (LRH) in the South-West, Ndop District Hospital (NDH) in the North-West, and Abong-Mbang District Hospital (ADH) in the Eastern Region of Cameroon. These facilities provide comprehensive healthcare services, including HIV testing and treatment and were purposefully selected for inclusion of urban, semi-urban, and rural populations.

### 2.3 Study period and population

The ASPA study was implemented at LRH from July through December 2015, and from June through November 2016 at the 2 other sites. The study population in the tPITC group consisted of parents living with HIV/AIDS receiving care in the hospital and their children of unknown HIV status, aged 6 weeks to 19 years. Similarly, in the bPITC group, the study population consisted of parents/guardians and their sick children of the same age group who attended the hospital outpatient department for any reason. Children or parents critically ill (in vital distress) were excluded from the study. For this article the study population was limited to children.

### 2.4 Eligibility criteria

Were eligible to participate, children and adolescents of unknown HIV status, aged 6 weeks to 19 years, and enrolled in the study through parents in HIV care (tPITC group) or parents/guardians accompanying them to the hospital (bPITC group).

### 2.5 Sample size

The sample size was that of the aforementioned ASPA study reported elsewhere.^[19]^

### 2.6 Study procedures

#### 2.6.1 Enrollment of participants and data collection

In the tPITC group, HIV-positive parents in care at the HIV treatment center (ART clinic) were counseled and invited by a trained counselor to participate in the study together with their children. These parents were offered a testing opportunity for their biological children in either the hospital or at home (community testing). In the bPITC group, parents/guardians were also counseled and invited to have their sick children tested for HIV irrespective of the reason of consultation.

In both groups, all parents/guardians who consented to participate in the study were enrolled together with their children. Pre-tested and structured questionnaires were used by a trained data clerk to collect socio-demographic information of participants. For children, we collected information on age, sex, educational level, and HIV testing history (previous HIV testing and result).

### 2.7 HIV testing and ART enrollment

For children younger than 18 months of age, HIV testing was performed using DNA-PCR techniques. For children aged 18 months and above, HIV testing was performed using 2 HIV antibody rapid tests according to the Cameroon national guidelines. During the study period, the WHO test and treat policy was not yet effective at the site level. Thus, children who tested HIV positive were assessed for ART eligibility using WHO clinical staging and/or baseline biological analysis, including CD4 count. Eligible children were initiated on ART and monitored according to the Cameroon national guidelines.

### 2.8 Outcomes measurement

#### 2.8.1 HIV case detection

Proportion of children/adolescents diagnosed for HIV infection.

#### 2.8.2 ART enrollment

Proportion of HIV+ children/adolescents enrolled on ART.

### 2.9 Data management and analysis

Anonymous data from the questionnaires were entered into a database and analyzed using STATA 2013 (College Station, TX: StataCorp LP). For the purpose of this article, we excluded from the ASPA dataset, all parents as well as children 15 years or above. Only children <15 years were included in this analysis because pediatric HIV age ranges from 0 to 14 years. The study outcomes were determined by computing and comparing the proportions using Chi-square or Fisher exact tests at 5% significant level.

### 2.10 Ethical considerations

Participation in the study was voluntary for both parents and children. Only parents who consented were enrolled and assent was requested from children above 11 years of age. Consent from parents was obtained via signed written consent form. Likewise, assent for children over the age of 11 years was obtained through a signed written assent form. The ASPA study received ethical approval from the Cameroon National Ethics Committee, the Ludwig-Maximilians-Universität, Munich (Germany) and the Albert Einstein College of Medicine (NY, USA). The ASPA study was also registered at clinicaltrial.gov (NCT03024762).^[19]^ This study was also approved by the Cameroon Ministry of Public Health.

## 3 Results

A total of 4719 children and adolescents were enrolled in the ASPA study. Six hundred forty adolescents who were 15 years of age or above were excluded from the dataset. Among children/adolescents included in this analysis (n = 4079), the age ranged from 6 weeks to 14 years, 51.8% were men, 49.6% had primary school level, 71.1% enrolled in the study through their mothers, and only 24.8% had previously tested for HIV (Table 1).

**Table 1:** Characteristics of children and adolescents enrolled in the study.

	N (4079)	% (95 CI)
Sex		
Female	1965	48.2 (46.6–49.7)
Male	2114	51.8 (50.3–53.4)
Age (months)		
<18	805	19.7 (18.5–21.0)
18–59	1169	28.7 (27.3–30.1)
60–119	1180	28.9 (27.6–30.3)
120–179	925	22.7 (21.4–24.0)
Education		
None	1544	37.9 (36.4–39.4)
Primary	2023	49.6 (48.1–51.1)
Secondary/higher level	512	12.6 (11.6–13.6)
Previously tested for HIV		
Yes	1011	24.8 (23.5–26.1)
No	3008	73.7 (72.4–75.1)
Unknown	60	1.5 (1.1–1.9)
Declared previous HIV test results		
Negative	979	24 (22.7–25.3)
Positive	9	0.2 (0.1–0.4)
Unknown	3091	75.8 (74.4–77.1)

The age disaggregation shows that 19.7% and 80.3% of enrolled children/adolescents were <18 and ≥18 months of age, respectively. These proportions correspond to children /adolescents eligible for PCR HIV testing (<18 months) and HIV serology (≥18 months), respectively. Overall, 3156 (77.4%) children/adolescents were tested for HIV. Of those, 22.4% and 77.6% (*P* < .001) were <18 months and ≥18 months, respectively. Among children/adolescents who tested for HIV, 98.1% (3095) received their results. Among those who did not receive their HIV test result (n = 61), 90.2% and 9.8% (*P* < .001) were children/adolescents <18 months and ≥18 months, respectively. Among children/adolescents who tested for HIV, 63 (2.0%) were found HIV+. Of those, 9 (14.3%) and 54(85.7%) (*P* = .24) were children <18 and ≥18 months, respectively. Among children/adolescents who tested HIV+, 48 (76.1%) were enrolled on ART. Children/adolescents <18 months and those ≥18 months contributed by 8.3% and 91.7% (*P* = .02), respectively to the total number of children/adolescents who enrolled on ART (Table 2).

**Table 2:** Contribution of children's age in HIV testing, case detection, and ART enrollment.

	Total n (col %)	Age <18 months	Age ≥18 months	
Outcome		n (row%)	n (row%)	*P* value^∗^
Children eligible for rapid antibody test				
No	805 (19.7)	805 (19.7)	0	N/A
Yes	3274 (80.3)	0	3274 (80.3)	
Children tested				
No	923 (22.6)	99 (10.7)	824 (89.3)	<.001
Yes	3156 (77.4)	706 (22.4)	2450 (77.6)	
Results received				
No	61 (1.9)	55 (90.2)	6 (9.8)	<.001
Yes	3095 (98.1)	651 (21.0)	2444 (79.0)	
Children's HIV results				
Negative	3032 (98.0)	642 (21.2)	2390 (78.8)	.24
Positive	63 (2.0)	9 (14.3)	54 (85.7)	
Children enrolled on ART				
No	15 (23.8)	5 (33.3)	10 (66.7)	.02
Yes	48 (76.1)	4 (8.3)	44 (91.7)	

## 4 Discussion

According to WHO, “efforts in the global research agenda on pediatric HIV must be focused on generating targeted evidence that improves HIV programme implementation through a better understanding of what works for infants and children.”^[20]^ Our study is a response to this need.

We found that the HIV testing uptake was statistically higher among children ≥18 months compared with those <18 months (77.6% vs 22.4%, *P* < .001). Indeed, among children enrolled in the study, those ≥18 months of age were 3-fold more likely to be tested for HIV compared with those <18 months. This was due to the fact that during the study period, the HIV antibody rapid tests were readily available in the laboratory, unlike the DBS-PCR testing kits that were affected by the recurrent stock outs during that same period. The stock outs of PCR testing commodities had been previously reported as factors affecting the early infant diagnosis (EID) of HIV among neonates in sub-Saharan Africa countries.^[21,22]^ The use of point-of-care (PoC) HIV testing technology could address this gap and improves the turn-around time of EID results as well as the early ART initiation among HIV infected neonates.^[23–25]^

Among children who tested for HIV, 98.1% received their results. Of those who did not receive results, the proportion of children <18 months were significantly higher compared with children ≥18 months (90.2% vs 9.8%, *P* < .001). This was mainly due to the long turned around time of EID PCR testing for children <18 months and the indeterminate HIV results for those ≥18 months. The use of PoC technology as indicated above and the availability of 3rd HIV rapid test (tie-breaker) in the laboratory should improve this outcome.

Among children who tested for HIV (n = 3156), 63 (2%) tested HIV+. Among those, children ≥18 months were predominantly represented compared with children <18 months (85.7% vs 14.3%, *P* = .24). Though this difference was not statistically significant, the HIV positivity among children ≥18 months was 6-fold higher compared with that of children <18 months. This suggests that a greater proportion of children living with HIV/AIDS are found among children ≥18 months. According to UNAIDS estimates, our finding is in line with the proportion of children <18 months living with HIV/AIDS in Cameroon and elsewhere.^[26]^ Therefore, adequate resources should be invested in testing children ≥18 months using HIV rapid tests.

Among children tested HIV+ (n = 63), 76.1% were enrolled on ART. Of those, the proportion of children ≥18 months was significantly higher compared with those <18 months (91.7% vs 8.3%, *P* = .02). Actually, the contribution of children ≥18 months in the pediatric ART cohort was 11-fold higher compared with children <18 months. This further demonstrates the need of adequate investments in case finding strategies among the subpopulation of children ≥18 months in Cameroon and probably beyond.

### 4.1 Policy implications

Our results show that children ≥18 months have the highest contribution with regards to pediatric HIV case load and ART enrollment. This indicates the role the rapid HIV test could play in bridging the current gap in pediatric HIV care. That notwithstanding, HIV testing for older children is still limited in most high burden pediatric HIV countries. In these countries, the most common entry point for HIV testing of children ≥18 months is the outpatient pediatric consultations or inpatient pediatric wards using the WHO recommended PITC.^[13]^ However, as indicated above PITC implementation had remained suboptimal across countries. As a result, only approximately 10% and 15% of HIV-infected young (15–24 years) men and women, respectively, in Sub-Saharan Africa are aware of their HIV status.^[27]^ This is a clear indication that many children ≥18 months are not adequately tested for HIV despite the higher HIV seropositivity among them as indicated by this study. This finding urges for a paradigm shift to give HIV testing for older children the adequate and proportionate attention and resources. This is a sine qua non condition to achieve the AIDS-free generation in high burden pediatric HIV countries.

We believe that in addition to all the barriers to the uptake of PITC reported to date^[28,29]^ the lack of adequate indicators to monitor the implementation of this strategy at facility level is a significant contributing factor to the current low uptake of PITC at facility level. Indeed, to monitor the performance of health facilities regarding HIV testing of their clients, it is important for these facilities to report both the number of outpatient consultations (denominator) and the number of patients tested for HIV (numerator). However, unlike the numerator, the denominator is most often not captured in the monthly summary HIV testing reports. This results to the lack of routine monitoring of health facilities performance with regards to HIV testing strategies, including PITC. On the other hand, other entry points for testing of older children (≥18 months) had not been adequately used by care providers for pediatric HIV case finding across countries in sub-Saharan Africa, including Cameroon. For example, though recommended by WHO since 2010,^[30]^ the uptake of index case HIV testing, using HIV infected parents in contact with health facilities (e.g., ART clinics) remains low in these countries. Recent studies from Kenya,^[31]^ Malawi,^[32]^ and Cameroon^[19,33,34]^ have demonstrated that index case testing is a high-yield strategy for pediatric HIV case finding. Thus, in addition of monitoring the denominator of clients visiting the health facilities, the adequate implementation of index case testing among children ≥18 months should also contribute significantly to improving ART expansion among children.

### 4.2 Strengths and limitations

To the best of our knowledge, this is the first analysis from primary data examining the contribution of the PCR HIV testing technology against the rapid HIV antibody tests in pediatric HIV case detection and ART enrolment. Thus, our study has filled an important scientific gap in ART expansion among children. However, our study is limited by the fact that the sites were not randomly selected and therefore, our results may have been affected by selection bias. That notwithstanding, the external validity of our study is stronger as it was conducted in 3 hospitals from 3 different geographic locations covering urban, semi-urban, and rural populations in Cameroon. Hence, this study has provided new evidence that should guide the investments of the already scarce HIV funding in achieving greater impact in resource-limited settings.

### 4.3 Conclusions and recommendations

This study shows that 4 out of 5 children found HIV+ and over 90% of those enrolled on ART were children ≥18 months. Therefore, while rolling out the POC HIV testing technology for children below 18 months, committing adequate and proportionate resources in testing children above 18 months should be a smart investment. Translating this evidence into practice would require tweaking the data collection tools to ensure the routine monitoring of HIV testing performance of health facilities, but also expanding HIV testing to all high-yield entry points. These adjustments are necessary to reducing the current gap in pediatric HIV treatment, and to achieve the AIDS-free generation in Cameroon and beyond.

## Acknowledgments

The authors are thankful to the study participants and the research assistants at R4D International for their contributions in the study management and data acquisition.

## Author contributions

**Conceptualization:** Habakkuk A. Yumo.

**Data curation:** Habakkuk A. Yumo, Jackson Jr. N. Ndenkeh.

**Formal analysis:** Habakkuk A. Yumo, Jackson Jr. N. Ndenkeh.

**Funding acquisition:** Habakkuk A. Yumo.

**Investigation:** Habakkuk A. Yumo, Jackson Jr. N. Ndenkeh.

**Methodology:** Habakkuk A. Yumo, Marcus Beissner, Christopher Kuaban, Thomas Loscher.

**Project administration:** Habakkuk A. Yumo, Isidore Sieleunou, Denis N. Nsame, Pius B. Kuwoh.

**Resources:** Habakkuk A. Yumo.

**Supervision:** Habakkuk A. Yumo, Jackson Jr. N. Ndenkeh, Isidore Sieleunou, Denis N. Nsame, Pius B. Kuwoh, Marcus Beissner, Christopher Kuaban, Thomas Loscher.

**Validation:** Habakkuk A. Yumo, Jackson Jr. N. Ndenkeh, Isidore Sieleunou, Marcus Beissner, Christopher Kuaban, Thomas Loscher.

**Visualization:** Habakkuk A. Yumo, Jackson Jr. N. Ndenkeh.

**Writing – original draft:** Habakkuk A. Yumo.

**Writing – review & editing:** Habakkuk A. Yumo, Jackson Jr. N. Ndenkeh, Isidore Sieleunou, Denis N. Nsame, Pius B. Kuwoh, Marcus Beissner, Christopher Kuaban, Thomas Loscher.

## References

[1] UNAIDS. UNAIDS Data 2019 [Internet]. Geneva, Switzerland; 2019. Available at: https://www.unaids.org/sites/default/files/media_asset/2019-UNAIDS-data_en.pdf. Accessed November 25, 2020.

[2] Teasdale CA Abrams EJ Yuengling KA. Expansion and scale-up of HIV care and treatment services in four countries over ten years. PLoS One 2020;15:02. 10.1371/journal.pone.0231667 PMC7162457 32298331

[3] Ford N Calmy A Mills EJ. The first decade of antiretroviral therapy in Africa. Glob Health 2011;7:01. 10.1186/1744-8603-7-33 PMC3192657 21958478

[4] El-Sadr WM Harripersaud K Rabkin M. Reaching global HIV/AIDS goals: what got us here, won’t get us there. PLoS Med 2017;14:01. 10.1371/journal.pmed.1002421 PMC5675304 29112691

[5] UNAIDS. Fact sheet - World AIDS day 2019. Geneva, Switzerland; 2019. Available at: https://www.unaids.org/sites/default/files/media_asset/UNAIDS_FactSheet_en.pdf. Accessed November 25, 2020.

[6] UNAIDS. AIDSinfo. Country factsheets. Cameroon 2018; 2019. Available at: https://aidsinfo.unaids.org/. Accessed November 25, 2020.

[7] Sam-Agudu NA Folayan MO Ezeanolue EE. Seeking wider access to HIV testing for adolescents in sub-Saharan Africa. Pediatr Res 2016;79:838–45. 26882367 10.1038/pr.2016.28

[8] Msellati P Ateba Ndongo F Hejoaka F Nacro B. [Impediments to HIV testing in HIV-infected children and teenagers in Africa: look for them where they are!]. Med Sante Trop 2016;26:10–4. 26986242 10.1684/mst.2015.0519

[9] Eba PM Lim H. Reviewing independent access to HIV testing, counselling and treatment for adolescents in HIV-specific laws in sub-Saharan Africa: implications for the HIV response. J Int AIDS Soc 2017;20:02. 10.7448/IAS.20.1.21456 PMC5577701 28799324

[10] United States of America. Department of State. PEPFAR BLUEPRINT: Creating an AIDS-free GENERATION. The Office of the Global AIDS Coordinator; 2012. Available at: https://www.avac.org/sites/default/files/resource-files/PEPFAR%20Blueprint.pdf. Accessed December 2, 2020.

[11] Kellerman SE Ahmed S Feeley-Summerl T. Beyond PMTCT: keeping HIV exposed and positive children healthy and alive. AIDS Lond Engl 2013;27:S225–33. 10.1097/QAD.0000000000000107 PMC4087192 24361632

[12] WHO. Department of HIV/AIDS. Prevention of Mother to Child Transmission of HIV (PMTCT). Briefing Note; 2007. Available at: https://www.who.int/hiv/pub/toolkits/PMTCT%20HIV%20Dept%20brief%20Oct%2007.pdf. Accessed December 2, 2020.

[13] World Health Organization, Joint United Nations Programme on HIV/AIDS. Guidance on Provider-initiated HIV Testing and Counselling in Health Facilities. Geneva: World Health Organization; 2007.

[14] Ahmed S Kim MH Sugandhi N. Beyond early infant diagnosis: case finding strategies for identification of HIV-infected infants and children. AIDS Lond Engl 2013;27: suppl: S235–45. 10.1097/QAD.0000000000000099 PMC4122794 24361633

[15] Leon N Lewin S Mathews C. Implementing a provider-initiated testing and counselling (PITC) intervention in Cape town, South Africa: a process evaluation using the normalisation process model. Implement Sci IS 2013;8:07–14. 10.1186/1748-5908-8-97 PMC3765808 23972055

[16] Davies M-A Kalk E. Provider-initiated HIV testing and counselling for children. PLoS Med 2014;11:01. 10.1371/journal.pmed.1001650 PMC4035263 24866364

[17] Lallemant M Chang S Cohen R. Pediatric HIV—A neglected disease? N Engl J Med 2011;365:581–3. 21848457 10.1056/NEJMp1107275

[18] WHO. Consolidated guidelines on HIV testing services. Geneva; Switzerland; 2015. Available at: http://www.who.int/hiv/pub/guidelines/hiv-testing-services/en/. Accessed on May 15, 2020.

[19] Yumo HA Kuaban C Ajeh RA. Active case finding: comparison of the acceptability, feasibility and effectiveness of targeted versus blanket provider-initiated-testing and counseling of HIV among children and adolescents in Cameroon. BMC Pediatr 2018;18:01. 10.1186/s12887-018-1276-7 PMC6156944 30253758

[20] WHO. Research for an AIDS free generation: a global research agenda for paediatric HIV. Geneva, Switzerland; 2017. Available at: http://www.who.int/hiv/pub/toolkits/cipher-research-paediatric-hiv/en/. Accessed on December 2, 2020.

[21] Ciaranello AL Park J-E Ramirez-Avila L. Early infant HIV-1 diagnosis programs in resource-limited settings: opportunities for improved outcomes and more cost-effective interventions. BMC Med 2011;9:01. 10.1186/1741-7015-9-59 PMC3129310 21599888

[22] Anaba UC Sam-Agudu NA Ramadhani HO. Missed opportunities for early infant diagnosis of HIV in rural North-Central Nigeria: a cascade analysis from the INSPIRE MoMent study. PLoS ONE 2019;14:e0220616. 31365571 10.1371/journal.pone.0220616 PMC6668908

[23] Arora DR Maheshwari M Arora B. Rapid point-of-care testing for detection of HIV and clinical monitoring. ISRN AIDS 2013;2013:02–3. 10.1155/2013/287269 PMC3767371 24052887

[24] Spooner E Govender K Reddy T. Point-of-care HIV testing best practice for early infant diagnosis: an implementation study. BMC Public Health 2019;19:02. 10.1186/s12889-019-6990-z PMC6560857 31185962

[25] Bianchi F Cohn J Sacks E. Evaluation of a routine point-of-care intervention for early infant diagnosis of HIV: an observational study in eight African countries. Lancet HIV 2019;6:e373–81. 30987937 10.1016/S2352-3018(19)30033-5

[26] UNAIDS. National HIV estimates files. Available at: https://www.unaids.org/en/dataanalysis/datatools/spectrum-epp. Accessed on May 15, 2020.

[27] UNAIDS. Fact Sheet 2016. Global Statistics 2015. Available at: www.unaids.org/sites/default/files/media_asset/20150901_FactSheet_2015_en.pdf. Accessed on May 15, 2020.

[28] Marwa R Anaeli A. Perceived barriers toward provider-initiated HIV testing and counseling (PITC) in pediatric clinics: a qualitative study involving two Regional Hospitals in Dar-Es-Salaam, Tanzania. HIV AIDS (Auckl) 2020;12:141–50. 32280281 10.2147/HIV.S235818 PMC7127846

[29] Kranzer K Meghji J Bandason T. Barriers to provider-initiated testing and counselling for children in a high HIV prevalence setting: a mixed methods study. PLoS Med 2014;11:02–5. 10.1371/journal.pmed.1001649 PMC4035250 24866209

[30] World Health Organization and UNICEF. Policy requirements for HIV testing and counselling of infants and young children in health facilities; 2010. Available at: https://apps.who.int/iris/bitstream/handle/10665/44276/9789241599092_eng.pdf. Accessed May 15, 2020.

[31] Wagner AD Wachira CM Njuguna IN. Active referral of children of HIV-positive adults reveals high prevalence of undiagnosed HIV. J Acquir Immune Defic Syndr 2016;73:e83–9. 27846074 10.1097/QAI.0000000000001184 PMC5175406

[32] Ahmed S Sabelli RA Simon K. Index case finding facilitates identification and linkage to care of children and young persons living with HIV/AIDS in Malawi. Trop Med Int Health 2017;22:1021–9. 28544728 10.1111/tmi.12900 PMC5575466

[33] Penda CI Moukoko CEE Koum DK. Feasibility and utility of active case finding of HIV-infected children and adolescents by provider-initiated testing and counselling: evidence from the Laquintinie hospital in Douala, Cameroon. BMC Pediatr 2018;18:05. 10.1186/s12887-018-1235-3 PMC6090739 30075712

[34] Yumo HA Ajeh RA Beissner M. Effectiveness of symptom-based diagnostic HIV testing versus targeted and blanket provider-initiated testing and counseling among children and adolescents in Cameroon. PLoS ONE 2019;14:06. 10.1371/journal.pone.0214251 PMC6502453 31059507

